# “Pill-in-the-Pocket” Treatment of Propafenone Unmasks ECG Brugada Pattern in an Atrial Fibrillation Patient With a Common SCN5A R1193Q Polymorphism

**DOI:** 10.3389/fphys.2019.00353

**Published:** 2019-03-29

**Authors:** Linling Li, Yanfei Ruan, Nian Liu, Qianqian Zhao, Mengxia Zhang, Xin Li, Song Zuo, Jiang Le, Kui Wu, Rong Bai, Changsheng Ma

**Affiliations:** Department of Cardiology, Bejing Anzhen Hospital, Capital Medical University, Beijing, China

**Keywords:** Brugada syndrome, atrial fibrillation, *SCN5A*, polymorphism, propafenone

## Abstract

**Background:** “Pill-in-the-pocket” (PIP) treatment with type IC drugs for cardioversion of recent-onset atrial fibrillation (AF) has been recommended in guidelines. Major adverse effects have been often reported, and the underlying mechanisms are proposed to be associated with the genetic backgrounds.

**Methods and Results:** A male patient was treated with PIP approach (propafenone 600 mg.po) for the conversion of new onset AF. His symptoms got worse and referred to emergency room; ECG showed a typical Brugada syndrome (BrS) type I ECG pattern with sinus rhythm. Genetic screening identified a common SCN5A polymorphism R1193Q. Propafenone blockade of I_Na_ was studied in HEK293 cells expressed SCN5A R1193Q channel and WT channel using patch clamp techniques. There was no significant difference in peak current and steady-state gating parameters between R1193Q and WT at baseline. At clinically relevant concentration of 2 μmol/L propafenone, use-dependent block (UDB) of I_Na_ was more pronounced in R1193Q versus WT (44.2 ± 7.2 versus 24.8 ± 5.7% at the frequency of 2 Hz, *P* < 0.05); IC_50_ of UDB was 2.9 ± 0.7 μmol/L for R1193Q and 8.1 ± 1.8 μmol/L for WT, respectively. Propafenone produced more left shift of steady-state inactivation and slower recovery from inactivation in R1193Q compared with WT.

**Conclusion:** A common SCN5A polymorphism R1193Q enhances UDB by propafenone and predisposes the patients to drug-induced BrS with PIP treatment. Our data suggest that R1193Q polymorphism is likely to be a genetic marker for the major adverse effects associated with propafenone PIP approach for AF patients’ management. Ajmaline challenge to rule out the presence of BrS should be considered prior to propafenone PIP therapy in AF patients who are identified to have R1193Q polymorphism.

## Introduction

Single oral loading dose of propafenone or flecanide as “pill-in-the-pocket” (PIP) treatment for pharmacological cardioversion of recent-onset atrial fibrillation (AF) has been recommended in the guideline for management of patients with AF.(Kirchhof et al., 2016) This approach resolves the symptoms within 2–3 h and significantly decreases the visits to the emergency room and hospitalizations, while major adverse effects have been reported, including significant QRS wave widening, atrial flutter at a rapid ventricular rate, Brugada syndrome (BrS) ECG pattern, and sinus arrest (Alboni et al., 2004; Daniell, 2011). It has been proposed that underlying heart diseases, pharmacodynamics associated with drug metabolism and drug-drug interaction are responsible for these adverse effects (Alboni et al., 2004). Consequently, PIP is required to prescribe only if the administration of propafenone and flecainide has been proved safe in hospital as indicated in the guideline (Kirchhof et al., 2016). While recently it was reported that intravenous administration of flecainide or propafenone in patients with recent-onset AF in hospital does not predict adverse effects during PIP treatment,(Alboni et al., 2010) suggesting that it should be used with caution patient management for the use of PIP approach in the cardioversion of AF.

There is emerging data that genetic predisposing factors underlie sodium channel blockers associated adverse effects given the fact that SCN5A mutations were identified in the AF patients who were unmasked BrS by administration of type IC drugs for cardioversion (Savio-Galimberti and Darbar, 2014). Previously, we demonstrated that the biophysical diversity of SCN5A mutations is a major determinant of response to sodium channel blockers in patients with SCN5A mutations (Ruan et al., 2007, 2010). Here we reported a case with a common R1193Q SCN5A polymorphism, who presented type I BrS ECG after PIP treatment with propafenone. *In vitro* study found that this SCN5A polymorphism increases the sensitivity to propafenone block. Therefore, the present study showed that a common SCN5A polymorphism is likely responsible for the adverse events observed in AF patients treated with PIP approach.

## Materials and Methods

This study was carried out in accordance with the recommendations of the program of “Effects of SCN5A polymorphism R1193Q on the parameters of electrocardiogram in Chinese Han and the investigation of underlying mechanisms.” The protocol was approved by the Medical Ethics Committee of Beijing Anzhen Hospital.

### Genetic Screening

Genetic analysis was performed by screening of the open reading frame of the SCN5A, GPD1L, CACNA1C, CACNB2, SCN1B, SCN3B, SCN10A, KCNE3, and KCNH2 genes as we previously reported (Ruan et al., 2007, 2010).

### Site-Directed Mutagenesis and Transfection in HEK Cells

The SCN5A mutations were engineered into wild-type (WT) cDNA cloned in pcDNA3.1 (Invitrogen) and confirmed by sequence analysis. HEK 293 cells were transfected with equal amount of Na^+^ channel α-subunit and hβ1 by a lipofection procedure as previously described (Ruan et al., 2007, 2010).

### Patch Clamp Study

Membrane currents were measured using whole-cell patch clamp procedures with Axopatch 700B amplifiers (Axon Instruments, Foster City, CA, United States). All signals were low-pass filtered at 5 kHz (Digidata 1440A, Axon Instruments). Internal pipette solution contained (mmol/L) aspartic acid 50, CsCl 60, Na_2_-ATP 5, EGTA 11, HEPES 10, CaCl_2_ 1, and MgCl_2_ 1, with pH 7.4 adjusted with CsOH. External solutions consisted of (mmol/L) NaCl 130, CaCl_2_ 2, CsCl 5, MgCl_2_ 1.2, HEPES 10, and glucose 5 with pH 7.4 adjusted with CsOH. In experiments designed to measure the voltage dependence of activation, external Na^+^ was reduced to 30 mmol/L with *N*-methyl-glucamine used as a Na^+^ substitute. Stock solution (10 mM) of propafenone hydrochloride was prepared by dissolving the powdered drug in DMSO. Appropriate amounts of the stock solution were added to the extracellular solution to reach various drug concentrations. Recordings were made at room temperature. The detailed procedures of patch clamp were described previously (Ruan et al., 2007, 2010).

### Statistics

Pclamp9.2 (Axon Instruments, Foster City, CA, United States) and Origin8.5 (OriginLab, Northampton, MA, United States) were used for data acquisition and analysis. Data are presented as means ± SE. An unpaired Student’s *t*-test was used to compare means; *P* < 0.05 was considered statistically significant.

## Results

### Clinical Presentation

**FIGURE 1:**
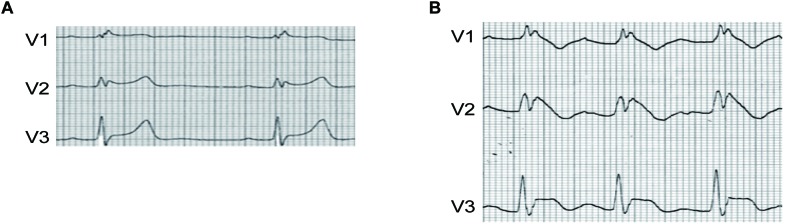
**(A)** ECG of the patient before PIP treatment with propafenone. **(B)** ECG of the patient after PIP treatment with propafenone. ST segment of V1–V3 was significantly elevated after PIP approach.

A 62-year-old man, who had a new onset of AF and no family history of syncope or cardiac death, was treated by intravenous propafenone (70 mg) for conversion of AF in emergency room. Sinus rhythm was resumed, and ECG showed incomplete right bundle branch block without ST-segment elevation in precordial leads (Figure 1A). He was discharged and given propafenone for PIP approach of recurrent AF management. Two weeks later, AF reoccurred, and he was self-treated with 600 mg propafenone orally. However, palpitation could not be resolved and he came into the emergency room. ECG revealed sinus rhythm with PR interval prolongation and significant ST-segment elevation in V1–V3, the type 1 BrS ECG pattern, as shown in Figure 1B. Six hours later, the ST-segment elevation in V1–V3 was completely resumed. BrS was suspected. Genetic screening was performed, and a heterozygous single-nucleotide transition (3578G > A) in SCN5A was identified, leading to a single amino acid replacement at position 1193 (R1193Q), this variant was identified in 50 references alleles from 374 ethnically matched controls.

### Gating Properties of R1193Q

**FIGURE 2:**
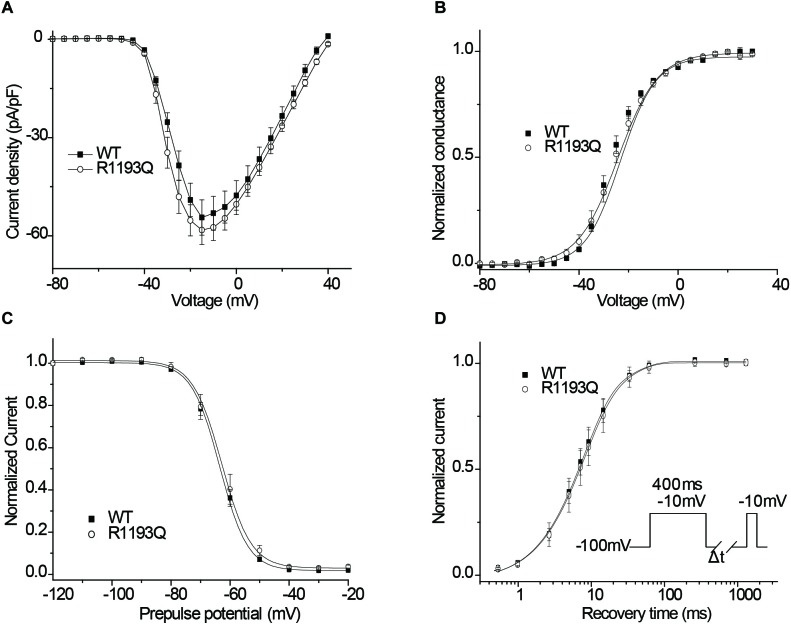
**(A)** Current–voltage relationship of peak currents. **(B)** The voltage dependence of SSA. **(C)** The voltage dependence of SSI. Experimental data were fitted with Boltzman relationships to obtain the parameters (Table 1). **(D)** Recovery from inactivation was assessed by the double-pulse protocol shown in inset and fitted using a biexponential function. Time constants and relative weights on averaged data are shown in Table 1.

As shown in Figure 2A, I_Na_ was activated by a voltage step from a holding potential of -100 mV to various test potentials. There was not substantially difference between WT and R1193Q in the current-voltage curves over a range from -80 to 40 mV; the peak current densities, measured at -10 mV, were comparable between WT and R1193Q (-58.24 ± 4.38 pA/pF versus -54.40 ± 5.36 pA/pF at -15 mV, *P* > 0.05, *n* = 10 cells for each group). Sustained sodium current (% of peak current) is not considerably increased in R1193Q compared with WT (0.44 ± 0.09% versus 0.34 ± 0.07%, *P* > 0.05, *n* = 10 cells for each group). No significant alterations were observed in SSA, SSI, and RFI between WT and R1193Q (Figure 2B–D and Table 1).

**Table 1:** Biophysical properties of R1193Q SCN5A channel.

	Steady-state activation			Steady-state inactivation			Recovery from inactivation		
	V_1/2_,mV	k,mV	n	V_1/2_,mV	k,mV	n	T_fast_, ms(%)	T_slow_, ms(%)	n
WT	-26.4 ± 0.4	6.7 ± 0.4	6	-63.4 ± 0.9	4.9 ± 0.1	7	9.6(87)	48.3(13)	9
R1193Q	-25.2 ± 0.2	7.6 ± 0.2	7	-62.8 ± 1.7	4.8 ± 0.2	8	9.7(81)	40.6(19)	10


*Because the functions for recovery from inactivation were fitted to the averaged data, error estimates on these parameters were not obtained*.

### Effects of Propafenone on Tonic Block and Use-Dependent Block

**FIGURE 3:**
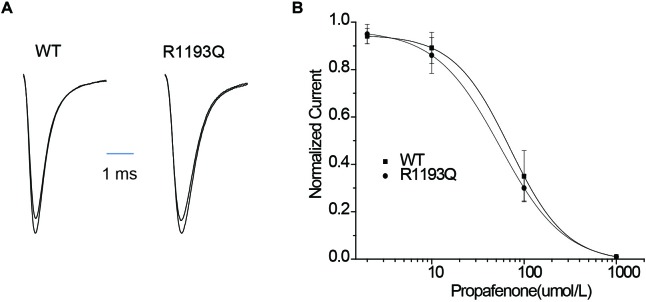
**(A)** Whole-cell current recordings of TB before and after propafenone perfusion (10 μmol/L). **(B)** Concentration dependence of TB by propafenone. Graph showed a peak current after drug application normalized to peak current in the absence of drug plotted against a function of drug concentration. IC50 values were 70.3 ± 9.3 μmol/L for WT (*n* = 6) and 54.9 ± 10.1 μmol/L for R1193Q (*n* = 6), respectively.

Class IC antiarrhythmic drugs may reveal the hidden BrS. Therefore, we characterized tonic block (TB) and use-dependent block (UDB) by propafenone in WT and R1193Q. TB can be measured by using infrequent pulse to assay sodium current in order to avoid extra UDB. Figure 3A shows I_Na_ recordings elicited by a 20-ms pulse before and after exposure to 10 μmol/L propafenone, TB of I_Na_ was compatible between in R1193Q versus WT (14.0 ± 4.2 versus 10.9 ± 3.7%, *n* = 8–9 cells for each group, *P* > 0.05). As shown in Figure 3B, IC50 values of TB were 70.3 ± 9.3 μmol/L for WT and 54.9 ± 10.1 μmol/L for R1193Q, respectively.

**FIGURE 4:**
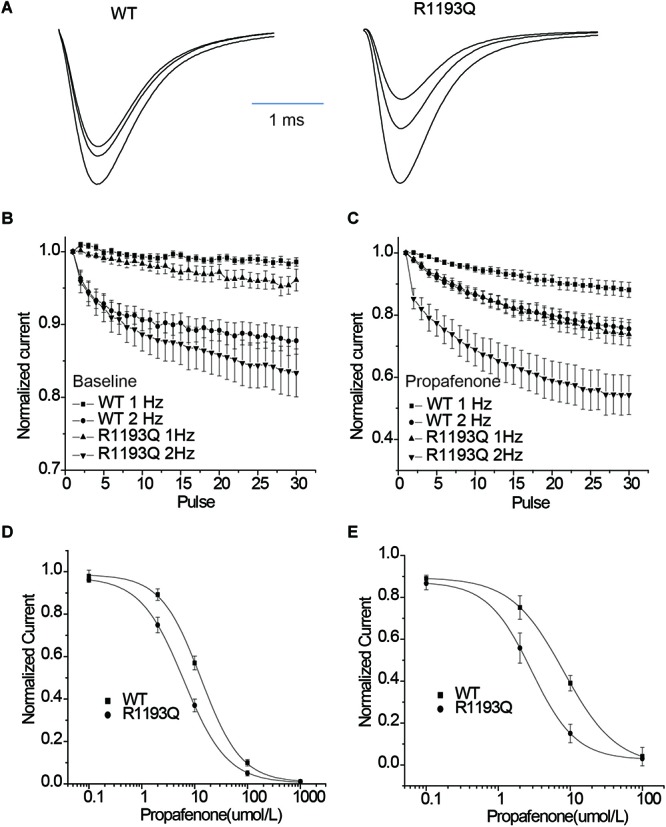
**(A)** Whole-cell current recordings of UDB showed representative current traces (1st, 15th, and 30th) that have been normalized to facilitate comparison of WT and R1193Q in the presence of propafenone (2 μmol/L). **(B)** Graph showed UDB for WT and R1193Q channel in the absence of propafenone at 1 and 2 Hz pacing. Plots of normalized peak Na^+^ current against the number of depolarizing pulse to –10 mV from a holding potential of –100 mV for 400 ms, *n* = 7 to 14 cells per condition. **(C)** Graph showed UDB for WT and R1193Q channel in the presence of propafenone (2 μmol/L) at 1 and 2 Hz pacing, *n* = 8–13 cells per condition. R1193Q exhibited more sensitive to propafenone; at 2 Hz pacing, the UDB of WT and R1193Q was 24.8 ± 3.5 and 44.2 ± 5.7%, respectively, *P* < 0.05. **(D)** Concentration dependence of UDB by propafenone at 1 Hz pacing, *n* = 6 for each concentration. IC50 values were 13.2 ± 2.6 μmol/L for WT and 6.2 ± 1.3 μmol/L for R1193Q. **(E)** Concentration dependence of UDB by propafenone at 2 Hz pacing, *n* = 6 for each concentration. IC50 values were 8.1 ± 1.8 μmol/L for WT and 2.9 ± 0.7 μmol/L for R1193Q.

Intriguingly, when tested the UDB by a protocol that mimicked the clinical condition by 400 ms depolarization from holding potential -100 mV, R1193Q presented more sensitive to propafenone blocking than WT despite without significant difference in UDB in the absence of propafenone (Figure 4A–C). In the presence of 2 μmol/L propafenone (clinically relevant concentration), there were more pronounced UDB in R1193Q versus WT (44.2 ± 7.2 versus 24.8 ± 5.7% at 2 Hz pacing, *P* < 0.01). At the frequency of 1 Hz, IC50 value for WT was 2.1 times higher than that for R1193Q (Figure 4D); at the frequency of 2 Hz, IC50 value for WT was 2.8 times higher than that for R1193Q (Figure 4E). Our data indicated that R1193Q significantly increased the channel response to propafenone at faster heart rate.

### Effect of Propafenone on Steady-State Inactivation

**FIGURE 5:**
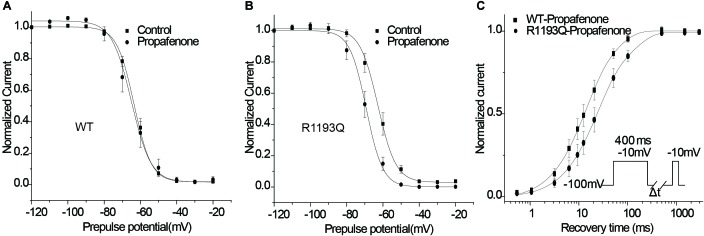
Effects of propafenone (2 μmol/L) on the channel gating properties. **(A)** Effect of propafenone on SSI in WT, propafenone shifted the V_1/2_ of SSI by -1.85 mV (from -63.41 ± 0.93 to -65.26 ± 2.61 mV, *n* = 7–10 cells for each group; *P* > 0.05). **(B)** Effect of propafenone on SSI in R1193Q, propafenone shifted the V_1/2_ by -6.96 mV (from -62.83 ± 1.72 to -69.79 ± 1.90 mV, *n* = 7–8 cells for each group, *P* < 0.05). **(C)** Effect of propafenone on time course of recovery from inactivation (protocol in inset), normalized current was plotted against a recovery interval (*n* = 8–10 cells for each group), time constants and relative weights on averaged data were as follows: for WT, T_fast_ = 12.0 ms, A_fast_ = 0.71, T_slow_ = 53.1 ms, A_slow_ = 0.29; for R1193Q, T_fast_ = 21.2 ms, A_fast_ = 0.70, T_slow_ = 123.5 ms, A_slow_ = 0.30.

According to the modulated receptor hypothesis, sodium channel blockers prefer to binding to the inactivated channel and tighter binding of a drug to the inactivated state must be accompanied by a shift in equilibrium from resting toward inactivated states once channels have bound drug. Thus, we compared the shift of SSI curves of WT and R1193Q in the presence of 2 μmol/L propafenone. As shown in Figure 5A,B, V_1/2_ of WT was shifted by -1.85 mV; while V_1/2_ of R1193Q was shifted by -6.96 mV. The augmented shift of V_1/2_ of SSI indicated that R1193Q induced stronger binding with propafenone than WT.

### Recovery From Propafenone Blockade

Use-dependent block is profoundly affected by the recovery from a drug block, and delayed recovery from sodium channel blockers has been implicated in the augmented drug sensitivity. Therefore, the recovery time course from propafenone block was evaluated. Cells were superfused with 2 μmol/L propafenone and depolarized to -10 for 400 ms from a holding potential of -100 mV, followed by a variable recovery time and a test pulse of -10 mV. As shown in Figure 5C, R1193Q recovered more slowly than WT in the presence of propafenone; the slower recovery led to fewer available channels and caused a decreased current when the R1193Q channels were repetitively activated by a train of depolarizing pulses.

## Discussion

Pill-in-the-pocket treatment with propafenone is recommended by the guideline for cardioversion of recent-onset AF (Kirchhof et al., 2016). In this study, we identified a SCN5A polymorphism R1193Q in a patient who presented type I BrS ECG after PIP treatment with propafenone. Patch clamp study revealed that R1193Q was sensitive to propafenone blocking and produced more significant UDB that was likely underlying the mechanism for the propefenone induced BrS.

R1193Q was originally described by Vatta et al. (2002) in a pair of affected Japanese dizygotic twins; one twin died unexpectedly during sleep at 4 months of age; the other twin had frequent VF episodes with typical BrS ECG; this variant was absent in 100 healthy control. Subsequently, R1193Q was reported on a series of SCN5A-associated channelopathies, such as long QT (LQT) syndrome, progressive cardiac conduction disease, overlap syndrome, and sudden infant death syndrome (Huang et al., 2006; Sun et al., 2008; Crotti et al., 2013). Several studies have characterized R1193Q gating properties *in vitro*. Wang et al. (2004) reported that it had a persistent sodium current and a shift of SSI toward more hyperpolarized voltages, thus accounting for the QTc prolongation and BrS phenotype, respectively; similar data were also obtained through the study of Huang et al. (2006). In the present study, R1193Q showed the tendency of an enhanced persistent sodium current and a left shift of SSI (0.6 mV), while there was no significant difference between R1193Q and WT. Our data were consistent with the study of Abdelsayed et al. (2015) in which R1193Q presented a tiny difference from WT. More recently, Abe et al. (2018) investigated the impact of various body temperature (25–38°C) on SCN5A WT channel and R1193Q channel; they found that R1193Q did not modulate NaV1.5 channel at various body temperature. Taken together, those studies demonstrated there was no significant abnormality of R1193Q channel that questions the pro-arrhythmic role of R1193Q channel.

A variety of drugs can unmask or induce phenotypes and arrhythmic manifestations of BrS, especially class IC drugs. Therefore, provocation test with sodium channel blockers has been recommended to the patients suspected of BrS but without a spontaneous type-1 Brugada ECG. The essential mechanism is mainly associated with BrS patients carried with “loss of function” mutation in SCN5A; sodium channel blockers would augment the gating defects of mutant SCN5A channel and predispose the patients to the phenotypes of BrS. Previously, we demonstrated that SCN5A mutation with a negative shift of SSI is more sensitive to sodium channel blocker (Ruan et al., 2007); subsequently, Makita et al. (2008) reported that negative shift of SSI represented common biophysical mechanisms underlying the phenotypic overlap of LQT3 and BrS. In the present study, R1193Q channel exhibited comparable SSI with WT, suggesting that other mechanisms are plausible. While in the presence of clinical concentration of 2 μmol/L propafenone, it produced pronounced UDB, especially at high pacing frequency; and slower recovery from SSI, we speculate that R1193Q channel likely increases the affinity for propafenone despite it locates at the intracellular loop between DII and DIII far away from traditional binding site for sodium channel blocker. Consistent with this hypothesis, we observed that propafenone also caused a more negative shift of SSI and a slower recovery from inactivation in R1193Q versus WT. This scenario as well occurred in a compound SCN5A mutation V232I+L1308F which identified in a patient with BrS phenotype induced by lidocaine; UDB by lidocaine was more pronounced in V232I+L1308F versus WT despite similar gating properties at baseline, the authors proposed that the compound SCN5A mutation increased the affinity of the cardiac sodium channel for lidocaine (Barajas-Martinez et al., 2008). For mimicking the action potential duration of human cardiac myocytes (Ruan et al., 2007), the protocol of depolarize to -10 mV for 400 ms was undertaken in the present study, which was different from other studies. Propafenone has been recognized as open and inactivated state sodium channel blocker (Edrich et al., 2005), prolongation of depolarization would increase the binding of propafenone to the sodium channel. Although R1193Q presented tiny abnormalities of gating properties difference from WT in the baseline, our protocol augmented these abnormalities in the present of propafenone, especially at fast pacing frequency. In a word, the pro-arrhythmic effect of R1193Q channel may be conditional, strong environmental stressors are required for unmasking arrhythmic phenotypes of individuals with a common SCN5A variant R1193Q.

Several studies found that R1193Q can be identified in healthy control with diverse prevalence among ethnic groups. For example, Ackerman et al. (2004) reported that the allele frequency of R1193Q was 8% in Asians, 0.3% in white, and no R1193Q was found in black and Hispanic healthy individuals. More recently, Matsusue et al. (2016) investigated more than 4000 DNA samples and found that R1193Q was detected in most Asian populations, but was sporadically observed or absent in European and African populations. In the present study, the allele frequency of R1193Q was 6.7%. Overall, those studies support that R1193Q is a relatively common polymorphism in Asian populations, but rare in other ethnic populations. Given the intrinsic elevated affinity for sodium channel blocks in R1193Q, the individual harboring SCN5A R1193Q variant would be high risk of pro-arrhythmic after exposure to sodium channel blocks.

Pill-in-the-pocket administration of propafenone has been recommended for self-treatment of PAF patients (Kirchhof et al., 2016). Although the patients had a tolerance of intravenous administration of flecainide or propafenone, 6% patients had major adverse events during PIP treatment in 11 months follow-up, including A-V block and sinus arrest, most of them occurred in the first oral treatment (Alboni et al., 2010), suggesting that some patients were more sensitive to sodium channel blockers. The proposed underlying mechanism is associated with the pharmacokinetic properties of propafenone which maybe interact with other drugs. The present study provided compelling evidence that the individual harboring a R1193Q variant would be at pro-arrhythmic risk when PIP treatment. The high prevalence of AF is observed among the patients with BrS. It is well known that the patients with symptomatic or asymptomatic BrS should avoid the exposure to sodium channel blockers since the patients with BrS usually harbor a rare SCN5A mutation with “loss of function.” Here, our study brings this concept to the next level by showing enhanced sodium channel block in a common polymorphism R1193Q, thus raising a warning flag to use PIP treatment in patients with SCN5A R1193Q.

It has been recognized that a common polymorphism of SCN5A S1103Y is a risk factor for arrhythmia, which has 10% allelic frequency in African Americans. Functional studies revealed that it had tiny channel dysfunctions and impaired cardiac repolarization reserve (Splawski et al., 2002). In general, it requires other environmental factor or “double hit” to cause arrhythmias (Giudicessi et al., 2018). Sun et al. (2011) found that S1103Y cardiac sodium channel variant was associated with ICD events in blacks with heart failure (hazard ratio:4.33). Based on the functional characterization of SCN5A R1193Q in the present study, we propose that SCN5A R1193Q is a risk factor for arrhythmia in Asian populations despite without evidence of epidemiological study. More recently, Makarawate et al. (2017) reported that SCN5A R1193Q polymorphism was associated with appropriate ICD shock therapy in symptomatic BrS patients with ICD treatment (hazard ratio:10.56) that is consistent with our hypothesis.

### Limitation

The present study reported a single case and did not evaluate the prevalence of BrS in a large cohort of AF patients harboring SCN5A after PIP treatment, while our results provided an evidence of R1193Q enhanced the sensitivity to propafenone, further clinical and epidemiological studies are warranted.

## Conclusion

We identified a common SCN5A R1193Q polymorphism in an AF patient in whom BrS ECG pattern was unmasked after PIP treatment of propafenone. *In vitro* studies elucidated enhanced sensitivity of R1193Q to propafenone as the underlying mechanism. These data suggest that caution should be used when AF patients with R1193Q variant receive propafenone PIP administration as this polymorphism is likely to be a genetic marker for the major adverse effects. In order to avoid potentially life-threatening arrhythmic event associated with propafenone PIP therapy, it is crucial to perform ajmaline challenge to rule out concealed BrS in AF patients carrying with R1193Q polymorphism.

## Author Contributions

NL, RB, and CM designed the study. YR, QZ, MZ, and SZ acquired the clinical data. LL, YR, and XL analyzed and interpreted the data. LL, YR, KW, and JL made the cell model. LL, YR, and NL recorded membrane currents. LL and NL summarized and analyzed the currents data. LL, YR, NL, and RB wrote the manuscript with input from all authors.

## Conflict of Interest Statement

The authors declare that the research was conducted in the absence of any commercial or financial relationships that could be construed as a potential conflict of interest.
